# Correction to: A Single Arabidopsis Gene Encodes Two Differentially Targeted Geranylgeranyl Diphosphate Synthase Isoforms

**DOI:** 10.1093/plphys/kiac119

**Published:** 2022-03-25

**Authors:** M Águila Ruiz-Sola, M Victoria Barja, David Manzano, Briardo Llorente, Bert Schipper, Jules Beekwilder, Manuel Rodriguez-Concepcion

*Plant Physiology*, https://doi.org/10.1104/pp.16.01392

In the originally published version of this manuscript, the funding acknowledgement was incomplete. The full acknowledgement should read as follows:

This work was funded by FEDER and Spanish Ministerio de Economía y Competitividad (grants BES-2009-025359, BIO2011-23680, BIO2014-59092-P, PCIN-2015-103, BIO2015-71703-REDT, and FPDI-2013-018882), Ministerio de Educación, Cultura y Deporte (FPU14/05142), European Commission H2020 ERA-IB-2 program (BioProMo), and Generalitat de Catalunya (2014SGR-1434 and 2015FI_B 00007).

These details have been corrected only in this correction notice to preserve the published version of record.

